# Case report of distal gastrectomy applied in primary duodenal bulb adenocarcinoma

**DOI:** 10.3389/fsurg.2025.1599159

**Published:** 2025-10-10

**Authors:** Jin Xiang, Yuan Li, Biao Zheng, Qingqun Yi

**Affiliations:** 1 Department of Gastrointestinal Surgery, Liuzhou Municipal Liutie Central Hospital, Liuzhou, Guangxi, China; 2 Department of Critical Care Medicine, Hunan University of Medicine General Hospital, Huaihua, Hunan, China

**Keywords:** duodenal bulb adenocarcinoma, distal gastrectomy, misdiagnosis, CAPEOX chemotherapy, case report

## Abstract

**Introduction:**

Primary duodenal bulb adenocarcinoma (PDA) is a rare and aggressive malignancy, frequently misdiagnosed due to its nonspecific clinical presentation and the lack of reliable biomarkers. While pancreaticoduodenectomy (PD) remains the standard radical treatment, its high complication rates have prompted the search for alternative therapeutic strategies.

**Methods:**

We report the case of a 51-year-old male presenting with recurrent gastrointestinal bleeding and severe anemia, initially misdiagnosed as a benign duodenal bulb ulcer perforation. After conservative management failed, the patient underwent laparoscopic distal gastrectomy with gastrojejunostomy. Postoperative pathological examination confirmed poorly differentiated adenocarcinoma (pT4N1M0) with intact mismatch repair proteins (MLH1/MSH2/MSH6/PMS2+). The patient completed 8 cycles of CapeOX chemotherapy and remained disease-free for 3 years.

**Results:**

This case highlights the diagnostic challenges associated with ulcer-mimicking PDA of the duodenal bulb. Distal gastrectomy achieved complete tumor resection with acceptable morbidity, suggesting its potential as a viable alternative to PD in carefully selected cases. The combination of surgical resection and CapeOX chemotherapy yielded favorable outcomes, although long-term efficacy requires further validation.

**Discussion:**

Clinicians should consider the possibility of malignant transformation in cases of medically refractory duodenal bulb ulcers. Distal gastrectomy combined with adjuvant chemotherapy may represent a feasible treatment option for locally advanced PDA, underscoring the need for additional studies to clarify its role in therapeutic algorithms.

## Introduction

Primary duodenal adenocarcinoma (PDA) is a malignant neoplasm arising from the glandular epithelium of the duodenum. This condition is relatively rare, especially in the duodenal bulb, with an estimated incidence of 0.04%–0.5% of all gastrointestinal tumors and 35%–45% of small intestine malignancies (1, 2). PDA predominantly affects middle-aged and elderly individuals, with no significant gender predilection. The disease is frequently associated with hereditary conditions such as familial adenomatous polyposis, hereditary nonpolyposis colorectal cancer, and Lynch syndrome (3, 4). Due to its rarity and nonspecific clinical presentation, PDA poses significant diagnostic and therapeutic challenges. The duodenum's anatomical proximity to the pancreas further complicates early detection, often resulting in diagnosis only after local invasion or distant metastasis has occurred.

## Case representation

**Figure 1:**
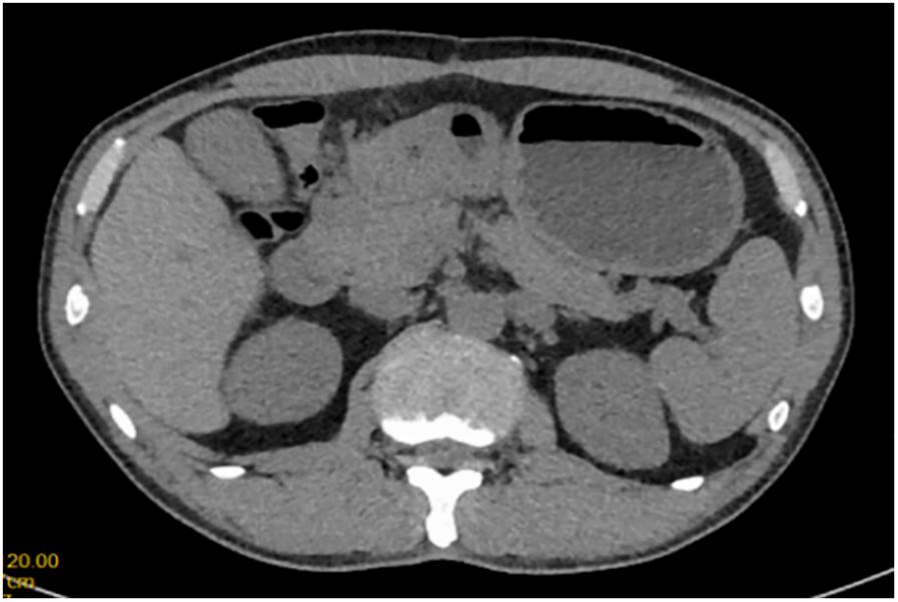
CT scan of duodenal bulb lesion.

**Figure 2:**
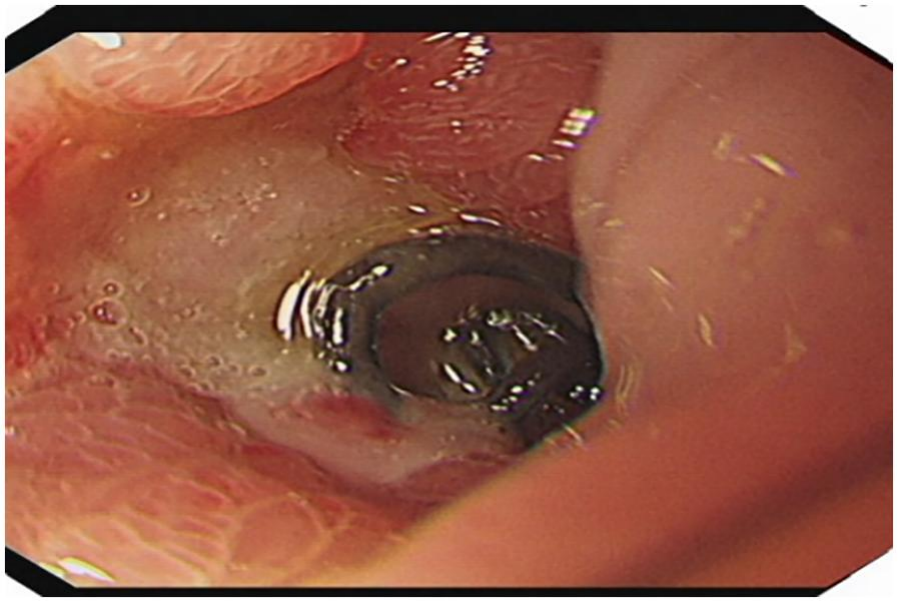
Duodenal bulb ulcer (after bulb perforation surgery).

**Figure 3:**
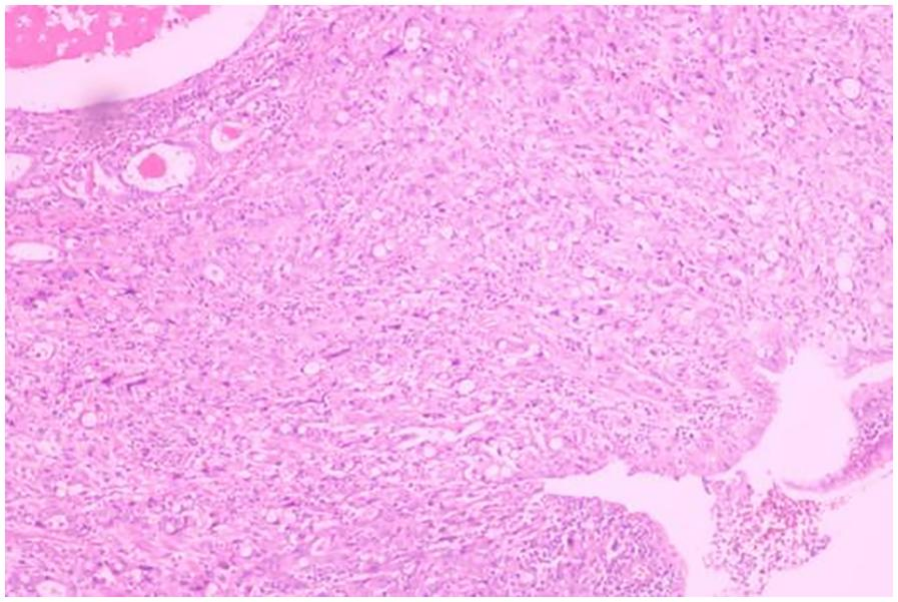
Poorly differentiated adenocarcinoma of the duodenal bulb, after distal gastrectomy (HE, ×40).

**Figure 4:**
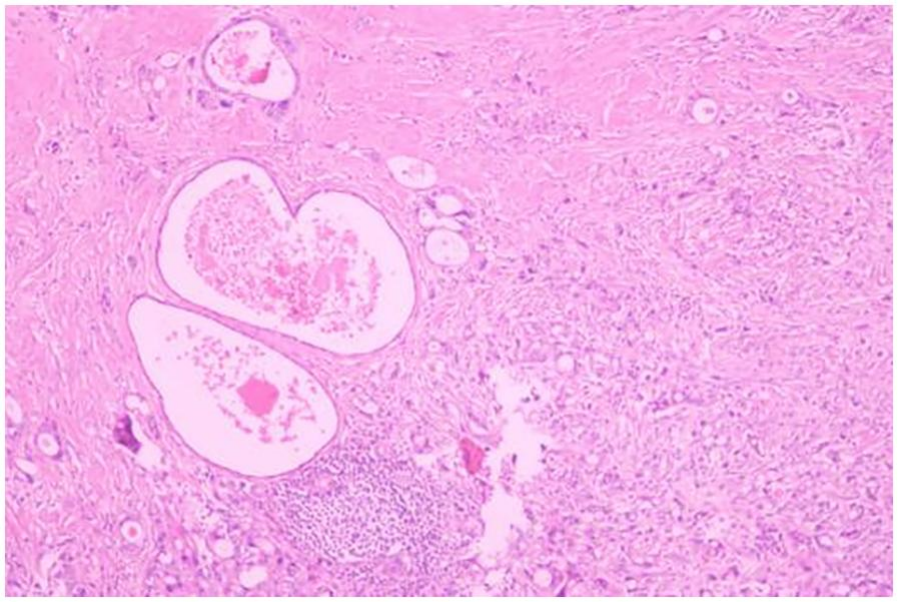
Poorly differentiated adenocarcinoma of the duodenal bulb, after distal gastrectomy (HE, ×40).

A 51-year-old male patient was admitted to the Department of Gastrointestinal Surgery in September 2020, presenting with “melena for one week and sudden abdominal pain for 11 h”. The patient had no significant medical history. Physical examination revealed right upper abdominal muscle tension, accompanied by tenderness and rebound pain in the same region. Laboratory tests indicated severe anemia (hemoglobin: 57 g/L), while tumor markers, including alpha-fetoprotein (AFP), carcinoembryonic antigen (CEA), carbohydrate antigen 199 (CA199), and carbohydrate antigen 724 (CA724), were within normal ranges. Computed tomography (CT) findings suggested gastrointestinal perforation. Consequently, the patient underwent surgical repair of a perforated duodenal bulb ulcer, followed by medical management with proton pump inhibitors. However, the patient failed to adhere to follow-up recommendations after discharge. In March 2021, the patient was readmitted due to “dizziness and fatigue for two days”. Laboratory tests revealed critically severe anemia (hemoglobin: 35 g/L). Gastroscopy demonstrated congested and erythematous gastric antral mucosa, pyloric deformity, and an ulcer on the anterior wall of the duodenal bulb with luminal narrowing, preventing endoscopic passage. Residual sutures and minor bleeding were observed (no biopsy was performed). Abdominal CT revealed thickening and irregularity of the gastric antrum and duodenal bulb walls, with no evidence of lymphadenopathy or masses (Figures 1, 2). Given the patient's recurrent duodenal bulb ulcer bleeding with stenosis and failure of conservative medical treatment, a laparoscopic distal gastrectomy (including resection of the bulb ulcer scar tissue) with gastrojejunostomy was performed. Postoperative pathological examination confirmed poorly differentiated adenocarcinoma in the anterior wall of the duodenal bulb, consistent with malignant transformation of the ulcer. The tumor, which measured 2.5 × 1.5 × 1.5 cm, is approximately 2 cm from the distal resection margin, with full-thickness invasion of the duodenal wall and extension into surrounding adipose tissue (Figures 3, 4). Intravascular cancer thrombi and perineural invasion were identified. Metastatic carcinoma was detected in six lymph nodes, and local mesenteric tissue infiltration was observed. Immunohistochemical staining results were as follows: CK7 (+), S-100 (nerve tissue +), CK20 (partially +), Ki67 (+ in approximately 45% of cells), Her-2 (−), and Villin (+). Mismatch repair proteins MLH1, MSH2, MSH6, and PMS2 were all expressed. Whole-body bone imaging showed no significant abnormalities. Postoperatively, the patient completed 8 cycles of CapeOX chemotherapy. Follow-up gastroscopy in September 2021 revealed multiple superficial ulcers in the isthmus and efferent loop. On May 13, 2024, follow-up gastroscopy identified an ulcer with stenosis in the isthmus (pathological biopsy confirmed no tumor recurrence), and abdominal enhanced CT showed no evidence of metastasis.

## Discussion and conclusions

Primary duodenal adenocarcinoma predominantly arises from the submucosal glands of the duodenum. Other rare types of duodenal malignancies include sarcoma, lymphoma, and carcinoid tumors. The majority of cases (approximately 85%) occur in the second, third, and fourth parts of the duodenum (5, 6), while tumors originating in the duodenal bulb are exceedingly rare. Additionally, the duodenum can serve as a site for metastatic spread from other malignancies, necessitating differentiation from cancers originating in the bile duct, pancreatic head, ampulla of Vater, and adjacent structures. Early symptoms of duodenal adenocarcinoma are often nonspecific, with common presentations including abdominal pain, nausea, vomiting, intestinal obstruction, gastrointestinal bleeding, and perforation (7, 8). Many patients initially present with dull or pre-meal abdominal pain, which can lead to misdiagnosis as gastritis or peptic ulcers, resulting in delayed treatment and disease progression. Tumors located near the duodenal papilla may present with jaundice. In this case, the patient exhibited recurrent gastrointestinal bleeding, culminating in severe anemia. Therefore, in patients unresponsive to medical therapy, the possibility of duodenal malignancy should be considered.

The diagnosis of duodenal cancer primarily relies on endoscopy and pathological biopsy (9, 10). However, early-stage duodenal bulb adenocarcinoma often lacks distinctive endoscopic features, making it difficult to differentiate from benign ulcers, which frequently leads to delayed diagnosis or misdiagnosis. Furthermore, due to the superficial nature of endoscopic biopsies, the false-negative rate of pathological examinations remains high. In recent years, advancements in diagnostic techniques, such as capsule endoscopy and double-balloon enteroscopy, have improved diagnostic accuracy (9). Duodenal hypotonic radiography can also aid in diagnosis, revealing rigid walls, reduced peristalsis, irregular niches, mucosal fold destruction, and luminal stenosis. While color Doppler ultrasound is useful for assessing vascular invasion, it is less effective for detecting tumors smaller than 2 cm. Abdominal enhanced CT and MRI have limited utility in identifying early duodenal tumors but play a significant role in surgical planning and clinical staging. MRI is particularly valuable for evaluating tumors in the duodenal papilla and their relationship with the bile duct and pancreas. Additionally, PET/CT may offer diagnostic value for primary malignant small bowel tumors and their metastase (11). In summary, duodenal cancer is challenging to detect in its early stages, and most cases are diagnosed only after the disease has advanced. This patient initially presented with gastrointestinal bleeding and severe anemia, having not undergone prior standardized medical treatment or gastrointestinal endoscopy, which contributed to the initial misdiagnosis as a benign duodenal bulb ulcer.

Currently, there are no specific tumor markers for duodenal cancer. However, some studies suggest that serum levels of carcinoembryonic antigen (CEA) and CA19-9 may serve as reference indicators for disease severity, postoperative recurrence, and metastasis, with elevated levels correlating with poorer survival rates (12, 13). Caudal type homeobox transcription factor 2 (CDX2), which is primarily expressed in the small intestine and colon, plays a key role in intestinal mucosal epithelial formation and differentiation. Patients with positive CDX2 expression tend to have a significantly better prognosis compared to those with negative expression (14, 15).

Surgical resection remains the most effective treatment for primary duodenal cancer, offering the best chance for improved survival. However, the choice of surgical approach, extent of resection, and lymph node dissection remain subjects of debate, with no standardized protocol established. The decision should be guided by factors such as tumor location, degree of malignancy, relationship with surrounding organs, tumor stage, the patient's overall condition, and the surgeon's expertise. Endoscopic mucosal resection (EMR) is suitable for intramucosal tumors, while radical surgery is the preferred approach for advanced duodenal cancer, potentially achieving clinical cure in some cases (16), Only when there is intramucosal disease and no significant lymph node metastasis, a modified operation such as distal gastrectomy or partial duodenectomy may be considered. Given that duodenal cancer often occurs in the descending portion and is closely associated with the pancreas and biliary system, pancreaticoduodenectomy (PD), also known as the Whipple procedure, is widely regarded as the standard for achieving radical resection (17–19). This procedure allows for complete tumor removal and comprehensive regional lymph node dissection, making it suitable for tumors in the duodenal bulb and the second portion of the duodenum. Some experts advocate for PD even in early-stage duodenal cancer and tumors located in the third and fourth portions of the duodenum, as it may offer a chance for clinical cure. Reported 5-year survival rates following radical resection of primary duodenal carcinoma (PDC) range from 37%–55% (1, 20). However, PD is a technically demanding procedure that requires highly skilled surgeons and advanced equipment. It involves multiple organs and is associated with a high rate of postoperative complications, including intestinal, pancreatic, and biliary fistulas, which occur in 5%–25% of cases. A study by Topal U shows that the most common postoperative complication was pancreatic fistula (33%), The average survival period of the patients was 40 months (21). The “Clinical Practice Guidelines for Duodenal Cancer 2021” in Japan note that there is insufficient evidence to support the prognostic benefit of lymph node dissection in duodenal cancer, and the patterns of lymph node metastasis around the duodenum remain poorly understood. In the first portion of duodenum, the lymph nodes in the subpyloric region (No. 6) and the posterior pancreatic head (No. 13) are considered to be sentinel lymph nodes, and lymphatic flow in the transvers and ascending part of the duodenum is speculated to flow from the inferior pancreatoduodenal artery and upper jejunal artery into the lymphatic system around the superior mesenteric artery. It is also possible that the preferred site of lymph node metastasis differs depending on localization. There is no evidence that shows that lymph node dissection for duodenal cancer contributes to a prolonged prognosis. The safety and efficacy of PD require further investigation. Some studies suggest that the 5-year survival rates for PD and partial duodenal resection are comparable. In a study involving 1,611 patients, 746 cases (46.3%) received simple resection treatment and 865 cases (53.7%) received radical resection treatment. Radical resection (e.g., in the form of pancreaticoduodenectomy) does not appear to impact survival compared with simple segmental resection for DA. Therefore, for advanced duodenal cancer, local duodenal resection with lymph node dissection may be considered, depending on tumor and patient-related factors. Therefore, it may be appropriate to choose a technique other than pancreato-duodenectomy, such as distal gastrectomy, local resection of the duodenum with lymph node dissection proximal to the tumor, for duodenal cancers that extend deeper than the submucosa, taking tumor and patient-related factors into full consideration. Limited resection should be considered as an alternative management option. Gastrectomy with duodenal bulb resection is rarely employed for duodenal tumors (22). In this case, the patient was initially misdiagnosed with a benign duodenal bulb ulcer, leading to the selection of distal gastrectomy. For malignancies in the duodenal bulb, distal gastrectomy may represent a viable surgical option, although further clinical data from larger studies are needed to validate this approach.

The role of adjuvant therapy following duodenal cancer surgery remains controversial. Most studies indicate that postoperative adjuvant therapy does not significantly improve overall survival. Japanese guidelines recommend against adjuvant therapy for resectable small intestine cancer (16), but advocate for microsatellite instability (MSI) testing. For unresectable or recurrent duodenal cancer, pembrolizumab monotherapy is recommended if high microsatellite instability (MSI-H) and DNA mismatch repair deficiency (dMMR) are detected. For advanced duodenal cancer, adjuvant chemotherapy regimens are often adapted from those used for gastric and colon cancers (23). Overman et al. found that FOLFOX and CAPOX regimens are commonly used as first-line chemotherapy for small intestine cancer. However, due to the lack of large-scale, multicenter randomized controlled trials, there is no standardized first-line chemotherapy regimen for advanced duodenal cancer.

In conclusion, malignant transformation of duodenal bulb ulcers is rare and difficult to distinguish from benign ulcerative lesions. Preoperative gastroduodenoscopy remains the primary diagnostic tool. For ulcerative lesions in the bulb that are unresponsive to medical treatment, the possibility of malignant transformation should be considered, and multiple endoscopic biopsies may be necessary for definitive diagnosis. A comprehensive treatment strategy centered on surgery is essential for optimizing patient outcomes.

## Data Availability

The datasets presented in this study can be found in online repositories. The names of the repository/repositories and accession number(s) can be found in the article/Supplementary Material.
